# The 17-gene Genomic Prostate Score assay as a predictor of biochemical recurrence in men with intermediate and high-risk prostate cancer

**DOI:** 10.1371/journal.pone.0273782

**Published:** 2022-09-01

**Authors:** Brian T. Helfand, Michael Paterakos, Chi-Hsiung Wang, Pooja Talaty, John Abran, John Bennett, David W. Hall, Amy Lehman, Tamer Aboushwareb

**Affiliations:** 1 NorthShore University HealthSystem, Evanston, Illinois, United States of America; 2 Exact Sciences Corporation, Madison, Wisconsin, United States of America; Carolina Urologic Research Center, UNITED STATES

## Abstract

The validated 17-gene Oncotype DX Genomic Prostate Score® (GPS™) assay risk-stratifies prostate-cancer patients with localized disease. The assay has primarily been utilized in lower risk patients deciding between active surveillance versus definitive therapy. In this retrospective cohort study, we analyze the association of the GPS result with time to biochemical recurrence post-prostatectomy in patients with National Comprehensive Cancer Network® (NCCN) intermediate and higher risk prostate cancer. The 141 patients included in the study were from the NorthShore University HealthSystem diagnosed 2014–2019 with NCCN intermediate (n = 109) or higher risk (n = 32) prostate cancer, treated with radical prostatectomy 2015–2019. The association of GPS result with time to biochemical recurrence was evaluated using univariable and multivariable Cox proportional hazards models in 120 patients with unfavorable intermediate or higher risk. Median (interquartile range) follow-up time was 28 (20 to 38) months. The GPS result was significantly associated with time to biochemical recurrence as both a continuous and dichotomous variable in univariable (hazard ratio [HR] per 20 GPS units 2.36, 95% CI 1.45–3.80, p < 0.001; HR for GPS result 41–100 vs 0–40 3.28, 95% CI 1.61–7.19, p < 0.001) and in multivariable models accounting for NCCN risk group (HR per 20 GPS units 2.14, 95% CI 1.31–3.46, p = 0.003; HR for GPS result 41–100 vs 0–40 3.00, 95% CI 1.43–6.72, p = 0.003) or biopsy Gleason Score and diagnostic PSA or PSA density. These results indicate that the GPS assay was a strong predictor of biochemical recurrence after radical prostatectomy in this unfavorable intermediate and higher risk prostate cancer patient population.

## Introduction

Accurate risk stratification of men with prostate cancer (PCa) is critical for choosing both the timing and type of treatment. The National Comprehensive Cancer Network® (NCCN) system uses various clinical factors, including tumor stage, Gleason pattern, grade group, PSA, and percent positive cores to stratify patients into very low, low, favorable intermediate (FIR), unfavorable intermediate (UIR), high, and very high risk groups [1]. Radiographic features are also used to aid risk stratification and diagnosis of PCa (e.g. Prostate Imaging Reporting and Data System (PI-RADS) for multiparametric MRI) [2]. In addition to clinical and radiographic factors [3–5], stratification can involve the use of biomarkers [6,7]. One currently available biomarker assay is the Exact Sciences (ES) Corporation 17-gene Oncotype DX Genomic Prostate Score® (GPS^TM^), which measures the expression of 12 cancer-related genes and 5 reference genes in PCa tissue obtained from needle biopsy [8]. The GPS assay is validated as an independent predictor of adverse pathology (AP) at radical prostatectomy (RP), biochemical recurrence (BCR) after RP, distant metastasis (DM) and PCa specific death (PCD) in men with NCCN® very low-, low- and intermediate-risk disease [9–12]. To date, the GPS assay has been most frequently used to help guide decision-making regarding active surveillance versus treatment with curative intent in NCCN FIR and lower risk patients. However, recent analyses suggest that the GPS assay may help stratify men with higher risk disease, namely those with NCCN UIR and high-risk PCa [13,14].

The recommendation to treat clinically higher risk men, particularly those with UIR and high-risk disease, is based in part on Level I evidence showing survival benefit from definitive therapy [1]. However, the intensity of treatment appropriate to this risk stratum remains uncertain. Prospective randomized clinical trials and retrospective series suggest that in addition to local control by surgery, radiation therapy in the form of external beam radiation, with or without hormonal therapy, with or without brachytherapy boost, and with or without hypofractionation could all successfully treat men in these risk categories [1,15,16]. While there is a significant range of potentially appropriate treatment intensities, current clinical, pathologic and radiographic variables are unable to reliably identify which patients can safely receive single modality therapy versus multi-modality therapy. Further risk stratification of men within NCCN UIR and high-risk groups would give more information, allowing patients and physicians more confidence in their decisions regarding treatment intensity.

Cullen et al. (2020) demonstrated that the GPS assay was able to stratify men with NCCN UIR PCa [14]. Specifically, NCCN UIR men who received GPS results 0–40 exhibited BCR rates that were indistinguishable from men in the NCCN favorable intermediate-risk (FIR) category. Further, UIR men with GPS 41–100 had BCR rates that were equivalent to men in the NCCN high-risk category. The study thus suggested that the GPS assay stratifies UIR men into those with high risk-like and those with FIR-like outcomes. The cut-off of 40 had been previously proposed to distinguish men with particularly poor prognosis in both unfavorable intermediate- and high-risk groups [10]. Together, these findings suggest that the GPS assay might stratify men in both UIR and high-risk categories.

To verify the findings of Cullen et al (2020) in UIR men, and to determine whether the GPS assay is prognostic in high-risk men, we conducted a blinded retrospective cohort study in an independent cohort. Specifically, we examined the relationship between the GPS result and outcomes for men with NCCN UIR and high-risk PCa.

## Methods

### Study design

The study was collaboratively and prospectively designed by researchers at NorthShore University HealthSystem (NorthShore) and ES. The study was approved by the NorthShore Institutional Review Board (study EH20-106, approved May 4, 2020), and all investigators agreed to the statistical analysis plan prior to analysis. An honest broker at NorthShore was responsible for maintaining the blind between the clinical and laboratory data until both data sources were locked.

### Patients

The study population consisted of all PCa patients between 18–75 years of age diagnosed with localized NCCN intermediate or higher risk PCa who were treated with RP at NorthShore between August 2015 and April 2019. Patients were required to have previously consented to inclusion in the NorthShore IRB-approved Urology Research Biobank, have tissue available from either diagnostic or confirmatory biopsy within 12 months of RP, and have no presence of Gleason pattern 5 in the biopsy samples.

### Pathology

For each patient, the biopsy specimen block containing the longest length of the highest available Gleason grade tumor was identified, graded independently by pathologists at both NorthShore and ES, and used to a perform a GPS assay. After independent scoring, disagreements were reconciled, and the consensus result was used as the Central Biopsy Score. If the block with the highest Gleason grade failed processing for any reason, testing was attempted on the second priority block. Blocks were chosen for processing per ES standard protocols.

### Endpoints

BCR was defined as either two successive post-RP PSA levels of ≥ 0.2 ng/mL, or initiation of salvage radiotherapy or hormonal therapy after a rising PSA. The BCR-free interval was defined as the time from diagnostic biopsy to BCR. Last date of contact with NorthShore, other primary cancers, or death prior to BCR were considered censoring events. The presence of DM was confirmed by CT, bone scan, MRI and/or PET/CT imaging. BCR, DM, and relevant clinical and pathologic data were extracted from electronic health records at NorthShore.

### GPS assay

The GPS assay measures the expression levels of 17 genes (12 cancer-related and 5 reference) in messenger RNA extracted from manually dissected tumor tissue from formalin-fixed prostate needle biopsies using quantitative reverse transcriptase polymerase chain reactions. The assay reports a GPS result scaled from 0 to 100 as a molecular measure of tumor aggressiveness, with higher values indicating more aggressive tumors [8,10]. It has been analytically and clinically validated as a significant independent predictor of multiple endpoints in men with newly diagnosed low and intermediate-risk PCa [9–11].

### Data analysis

Patient demographic and clinical characteristics were summarized using appropriate descriptive statistics. For the primary aim, Cox proportional hazards (PH) models were used to analyze the association between time to BCR and GPS result. Hazard ratios (HR) with 95% profile-likelihood confidence intervals (CI) were calculated. For the continuous GPS result, the HR was calculated per 20 units, as in previous studies [9–11,14,17]. The HR for dichotomous GPS was calculated for GPS values 41–100 versus 0–40, as used previously [17]. The likelihood ratio test was used, with two-sided p-values < 0.05 considered statistically significant. The PH and linearity assumptions were assessed for all models using methods described by Lin et al. (1993) [18]. An additional check for PH included the interaction term between each covariate and log(time) in the models. Due to the expected final sample size and number of BCR events, pre-planned multivariable analyses were restricted to the GPS result and NCCN risk group. There were no BCR events in the NCCN favorable intermediate subgroup, so all analyses involving the BCR endpoint were limited to patients with NCCN unfavorable intermediate or higher risk. In addition to the analysis for the entire cohort, a pre-specified analysis on the association of the GPS result with time to BCR was performed on the subpopulation of UIR patients only.

Univariable analyses were performed to identify the clinical factors associated with time to BCR to assess which variables warranted inclusion with the GPS result in multivariable models. Post-hoc exploratory analyses with the GPS result included adjustment for additional clinicopathologic covariates in separate multivariable models. Due to the number of BCR events in this cohort (n = 35), a decision was made prior to performing these analyses to include no more than 2 clinical covariates in addition to GPS result in any of the multivariable models. Therefore, several multivariable models are presented using different combinations of covariates from the set of covariates that had statistically significant univariable associations with time to BCR.

All analyses were performed in SAS version 9.4 for Windows (SAS Institute, Cary, NC) and R version 4.0.1 [19]. Graphics were created using the ggplot2 package [20].

## Results

### Patient characteristics

**Fig 1:**
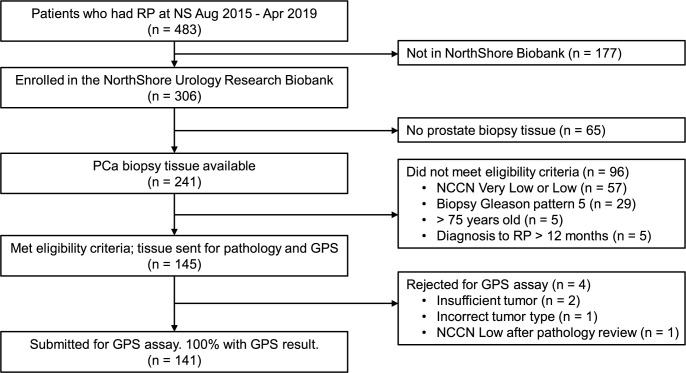
Flow diagram. Flow diagram showing included (n = 141) and excluded (n = 342) patients.

Of the 483 patients who were consented for biobanking and had RP between August 2015 and April 2019, 141 were included in the study (see Fig 1 for details about exclusions). All 141 samples submitted to the laboratory for the GPS assay returned a valid result. The characteristics of the final analytic cohort are shown in Table 1. There were 109 (77%) NCCN intermediate-risk (21 [15%] favorable and 88 [62%] unfavorable), 27 (19%) high-risk, and 5 (4%) very high-risk patients. The median GPS result was 39; 44% had a GPS result 41–100. There was a weak correlation between NCCN risk group and GPS result (Pearson’s *r* = 0.27, p = 0.001) (S1 Fig).

**Table 1:** Patient characteristics.

Age at diagnosis, yrs		Central Biopsy Gleason Score (GG), n (%)	
Median (IQR)	64 (57 to 68)	3+3 (GG 1)	1 (0.7%)
Range	44 to 75	3+4 (GG 2)	72 (51.1%)
Mean (SD)	62.57 (6.93)	4+3 (GG 3)	45 (31.9%)
< 65	77 (54.6%)	4+4 (GG 4)	23 (16.3%)
≥ 65	64 (45.4%)		
**Race**		**NCCN Risk Group, n (%)**	
Caucasian	111 (78.7%)	FIR	21 (14.9%)
African American	15 (10.6%)	UIR	88 (62.4%)
Asian	5 (3.5%)	High	27 (19.1%)
Other	10 (7.1%)	Very High	5 (3.5%)
**BMI, kg/m** ^**2**^		**GPS result**	
Median (IQR)	31.2 (27.9 to 34.7)	Median (IQR)	39 (30 to 48)
Range	20.8 to 51.9	0–40	79 (56%)
Mean (SD)	32.0 (6.5)	41–100	62 (44%)
**Diagnostic PSA, ng/mL**		**GPS result by NCCN Risk Group, median (IQR)**	
Median (IQR)	6.90 (4.69 to 10.50)	FIR	33 (26, 43)
Range	2.11 to 83.22	UIR	39 (30.5, 48)
Mean (SD)	9.95 (10.16)	High	40 (27, 51)
		Very High	58 (50, 60)
**PSA Density, ng/mL** ^**2**^		**Follow-up**	
Median (IQR)	0.23 (0.16 to 0.41)	Median time, mos (IQR)	26 (17 to 35)
Range	0.07 to 2.79	Total (%) with BCR	35 (25%)
Mean (SD)	0.35 (0.38)	Total (%) with DM	8 (5.7%)
**Clinical T-stage, n (%)**			
cT1c	111 (78.7%)		
cT2a	15 (10.6%)		
cT2b	9 (6.4%)		
cT2b/c	1 (0.7%)		
cT2c	5 (3.5%)		

Characteristics of patient cohort (n = 141).

BCR = biochemical recurrence, BMI = body mass index, DM = distant metastasis, FIR = favorable intermediate risk, GG = Grade Group, IQR = interquartile range, NCCN = National Comprehensive Cancer Network, PCa = prostate cancer, PSA = prostate-specific antigen, SD = standard deviation, UIR = unfavorable intermediate risk.

BCR occurred in 35 patients (25%), 8 were diagnosed with DM via imaging, and none experienced PCa-related mortality. All 8 patients with DM had GPS results above the median, with a range between 40 and 58. Median (IQR) follow-up time for the entire cohort was 25 (17 to 35) months; for patients who did not experience BCR it was 28 (20 to 38) months.

### Univariable results

**Fig 2:**
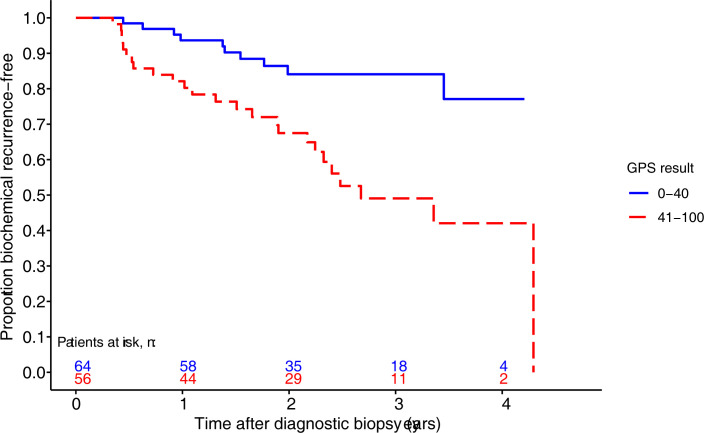
Kaplan-Meier curves stratified by GPS result. Kaplan-Meier curves showing the proportion of patients remaining free of biochemical recurrence from time of biopsy stratified by GPS result 0–40 and 41–100 (n = 120).

The 35 BCR events occurred in patients with NCCN unfavorable intermediate, high, or very high risk disease. All analyses on the BCR endpoint are limited to patients in these risk groups (n = 120). The GPS assay was significantly associated with time to post-biopsy BCR (Table 2), whether considered as a continuous or dichotomous predictor. Fig 2 shows the Kaplan-Meier plot for the proportion remaining BCR-free, stratified by dichotomous GPS result.

**Table 2:** Hazard ratios in univariable models (n = 120).

Variable	Events / n	HR	95% CI	p-value
GPS result				
GPS result per 20-unit increase	35 / 120	2.36	1.45 to 3.80	< .001
GPS result				< .001
0–40	10 / 64	1.00 (ref)		
41–100	25 / 56	3.28	1.61 to 7.19	
Clinical/demographic/pathologic: continuous^a^				
Age (yrs) at diagnosis	35 / 120	1.17	0.81 to 1.73	0.402
BMI (kg/m^2^)	35 / 120	0.81	0.56 to 1.14	0.240
Diagnostic PSA (ng/mL)	35 / 120	1.60	1.22 to 2.00	0.001
% positive cores	35 / 120	1.16	0.82 to 1.66	0.393
PSA density (ng/mL^2^)	35 / 120	1.60	1.24 to 1.97	< .001
Clinical/demographic/pathologic: categorical				
Age (yrs) at diagnosis				0.202
< 65	15 / 64	1.00 (ref)		
≥ 65	20 / 56	1.55	0.79 to 3.10	
BMI (kg/m^2^)				0.604
< 25	6 / 16	1.00 (ref)		
25 - < 30	7 / 29	0.56	0.17 to 1.80	
≥ 30	22 / 75	0.76	0.32 to 2.06	
Biopsy grade group^b^				0.021
2	9 / 51	1.00 (ref)		
3	16 / 45	2.22	0.98 to 5.49	
4	10 / 23	3.56	1.40 to 9.34	
Clinical stage				0.517
T1c	26 / 91	1.00 (ref)		
T2a	3 / 14	0.51	0.08 to 1.71	
T2b/c	6 / 15	1.21	0.45 to 2.77	
NCCN risk group				0.006
Unfavorable Intermediate	22 / 88	1.00 (ref)		
High	9 / 27	1.80	0.78 to 3.81	
Very High	4 / 5	8.39	2.40 to 22.76	
PSA density (ng/mL^2^)				0.352
≥ 0.15	5 / 22	1.00 (ref)		
< 0.15	30 / 98	1.54	0.65 to 4.52	

Hazard ratios (HR) from univariable Cox proportional hazards regression models on the association of the GPS result and various clinical, demographic, and pathologic variables with time to post-biopsy BCR in patients with NCCN unfavorable intermediate or higher risk prostate cancer (n = 120).

^a^ HR is for a one standard deviation (SD) increase. See Table 1 for SD values.

^b^ n = 119, excluding one patient with biopsy Grade Group 1.

BMI = body mass index, GPS = Genomic Prostate Score, HR = hazard ratio, PSA = prostate-specific antigen.

**Fig 3:**
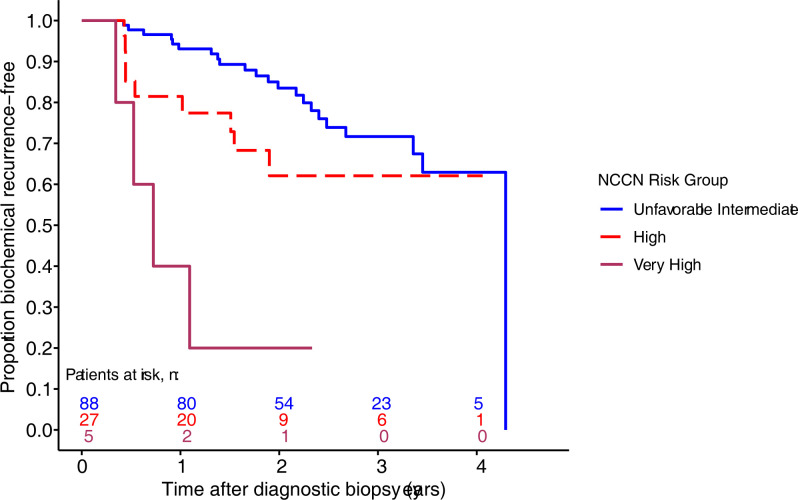
Kaplan-Meier curves stratified by NCCN risk group (n = 120). Kaplan-Meier curves showing the proportion of patients remaining free of biochemical recurrence from time of biopsy stratified by NCCN risk group.

Results from univariable Cox PH models indicate that several clinical, demographic, and pathologic variables are predictors of BCR (Table 2). NCCN risk group was associated with time to BCR; Kaplan-Meier plots for this variable are shown in Fig 3. Other variables with statistically significant associations with time to BCR included diagnostic PSA, PSA density, and biopsy Gleason Score (Table 2).

### Multivariable results

Multivariable analyses included the GPS result and NCCN risk group as pre-specified, and the predictive variables identified from the post-hoc univariable models (Table 2). In all models, the GPS result remained a significant predictor of time to BCR (Table 3). Importantly, for the pre-specified analysis, the GPS result was a strong predictor of time to BCR independent of NCCN risk grouping, as both a continuous variable and a dichotomous variable (Table 3, Models 1 and 4). All HR estimates from the multivariable models are presented in S1 Table.

**Table 3:** Hazard ratios in multivariable models (n = 120).

Model	HR for GPS	95% CI	p-value
**Continuous GPS result**			
1: GPS result per 20-unit increase, NCCN risk group	2.14	1.31 to 3.46	0.003
2: GPS result per 20-unit increase, diagnostic PSA, biopsy GS	2.28	1.35 to 3.82	0.002
3: GPS result per 20-unit increase, PSA density, biopsy GS	2.15	1.29 to 3.57	0.004
**Dichotomous GPS result**			
4: GPS result 41–100 vs 0–40), NCCN risk group	3.00	1.43 to 6.72	0.003
5: GPS result (41–100 vs 0–40), diagnostic PSA, biopsy GS	2.84	1.36 to 6.35	0.005
6: GPS result (41–100 vs 0–40), PSA density, biopsy GS	3.00	1.44 to 6.70	0.003

Hazard ratios (HR) for the GPS result and time to post-biopsy BCR in multivariable Cox proportional hazards models, after adjusting for clinical and pathologic covariates (n = 120).

BCR = biochemical recurrence; GPS = Genomic Prostate Score; GS = Gleason Score; NCCN = National Comprehensive Cancer Network; PSA = prostate-specific antigen; RP = radical prostatectomy.

### Subgroup analysis results

For the pre-specified analysis of the association of the GPS result with time to BCR in the NCCN UIR subpopulation (n = 88 patients, 22 with BCR), the HR for a 20-unit increase was 1.68 (95% CI: 0.85 to 3.21; p = 0.135), and the HR for 41–100 versus 0–40 was 2.2 (95% CI: 0.93 to 5.55; p = 0.074). While these results were not statistically significant, likely due to modest sample size, the estimated hazard ratios and survival curves suggest that the GPS is prognostic in this subgroup (S2 Fig).

All PH models use time from biopsy to align with previous work^7,11^. Sensitivity analyses using time from RP rather than biopsy to BCR provided similar results, which are presented in the Supplementary Materials (S2 and S3 Tables, S3 and S4 Figs).

## Discussion

Men who have clinically higher risk disease, but with localized PCa face challenging treatment decisions, with options including active surveillance (for FIR and sometimes UIR patients), RP, external beam radiation therapy, and androgen deprivation therapy of variable duration [21]. Therapies can be used alone (monotherapy) or in combinations (multimodal therapy), and each has different expected benefits and adverse effects. Various clinical and pathologic features are used to stratify men into particular NCCN (or other) risk categories, which then inform the decision-making process, with higher risk cancers treated more aggressively [1]. For example, the presence of Gleason pattern 5, indicative of very aggressive disease, is usually treated with multimodal therapy, which is in part why we did not include such patients in this study. However, within clinicopathologic risk groups, there can be a wide range of disease aggressiveness [22]. Our finding of a wide range of GPS values among patients in this cohort is consistent with this observation.

The data presented in this study indicate that a GPS result could be used to further risk-stratify UIR/high-risk patients. The GPS assay is shown to be an independent predictor of BCR in a cohort of 120 men with unfavorable intermediate- and higher risk PCa. As in the previous study [14], the difference in prognosis for patients with high versus low values of the GPS result is substantial in this cohort of intermediate- to high-risk patients. For the analysis of UIR patients only, the GPS result was not a statistically significant predictor of BCR, though the BCR-free survival curves are suggestive (S2 Fig), perhaps due to the relatively low number of patients (n = 88) and BCR events (n = 22) in this subpopulation. Though it was not a primary objective of the study, all 8 of the patients experiencing DM within the time frame of this study had GPS values ≥40 (range 40–58), suggesting that the GPS assay may be prognostic for this outcome in this higher risk population.

The data presented in this study provide additional evidence that the GPS assay may provide an important tool for the physician and patient to confidently choose a management plan. Patients with low GPS results might be advised to consider monotherapies while more aggressive multimodal therapies could be considered for patients with higher GPS results. Shared decision making is an integral part of the disease management strategy; the more information available to patients and their physicians during treatment planning, the better the outcomes [23,24].

We note that the cut-point of 40 should be regarded in a similar manner to other cut-points for continuous clinical variables. For example, PSA values of 10 and 20 are used in NCCN risk stratification. However, an intermediate-risk patient with a PSA of 11 is likely to be treated similarly to a low-risk patient with a PSA of 9, all else being equal. Likewise for GPS: though the cut-point of 40 clearly separates the two groups of patients as a whole in terms of risk of BCR, individuals with a GPS result close to 40, whether above or below, should be counseled and treated similarly, with all other available data carefully taken into consideration.

Strengths of the study include using a contemporary cohort treated with RP, which included UIR and HR patients. GPS assays were performed on biopsies taken within 12 months of RP, pathologic findings were independently confirmed at two sites, and pathology reporting was blind to GPS result and vice-versa. In addition, clinical covariates of interest were available for all subjects. Limitations of the study include the single institution, retrospective design as well as the fact that all patients were treated initially with RP, which may limit the utility of the results when employing other definitive therapy methods. We note that consent for inclusion into the NorthShore biobank occurred at the time of radical prostatectomy surgery. As such, in addition to demographic variables such as race, a patient’s biopsy-based risk may have influenced consent decisions. While it is unclear whether such biases would alter our findings, it is a possibility that should be considered.

BCR was the primary endpoint; however, larger studies investigating the GPS assay for predicting DM and PCa-related mortality are also needed in this population. Finally, our relatively modest sample size limited our multivariable modeling to only three variables at a time. However, we did include NCCN risk group, which does capture many clinical and pathologic variables of interest [21].

In conclusion, this study has shown that the GPS assay is a strong independent predictor of BCR in a population of patients with NCCN unfavorable intermediate- and higher risk disease. The poor outcomes of surgically treated men with GPS results 41–100 in this study suggest that physicians may consider more aggressive treatment for such patients while allowing for a de-escalation of therapy for those with a GPS result 0–40. Future studies should assess the GPS assay’s association with outcomes after radiotherapy, with or without hormonal therapies, as well as the clinical impact of mono versus multimodal therapies in higher genomic risk patients.

## Supporting information

S1 Fig Distribution of GPS results by NCCN risk group (n = 141). The median GPS result increased with higher NCCN risk group (Table 1 of manuscript), though the overall correlation between GPS and NCCN risk group was modest: r = 0.27 (p = 0.001). There was a broad and overlapping range of GPS results between the risk groups, with the exception of Very High, which had only 5 patients. This modest correlation has been observed in prior cohorts (see Cullen J, Rosner IL, Brand TC, et al. A Biopsy-based 17-gene Genomic Prostate Score Predicts Recurrence After Radical Prostatectomy and Adverse Surgical Pathology in a Racially Diverse Population of Men with Clinically Low- and Intermediate-risk Prostate Cancer. Eur Urol. 2015 Jul;68(1):123–31).
(EPS)

S2 Fig Kaplan-Meier curves stratified by GPS result for UIR patients (n = 88). Kaplan-Meier curves showing the proportion of NCCN unfavorable intermediate risk patients remaining free of biochemical recurrence from time of prostate cancer diagnosis stratified by GPS result >40 and ≤40. The difference in the two curves is not significant, likely due to modest sample size, but the curves and the estimated hazard ratios (see Results) suggest that the GPS may be prognostic in this subgroup.
(EPS)

S3 Fig Kaplan-Meier curves (time from RP) stratified by GPS result for patients with unfavorable intermediate, high, and very high risk (n = 120). Kaplan-Meier curves showing the proportion of patients remaining free of biochemical recurrence from time of prostatectomy stratified by GPS result 0–40 and 41–100. These results are essentially the same as for BCR calculated from time of biopsy/diagnosis (see Fig 2).
(EPS)

S4 Fig Kaplan-Meier curves (time from RP) stratified by NCCN risk group for patients with unfavorable intermediate, high, and very high risk (n = 120). Kaplan-Meier curves showing the proportion of patients remaining free of biochemical recurrence from time of prostatectomy stratified by NCCN risk group. These results are essentially the same as for BCR calculated from time of biopsy/diagnosis (see Fig 3).
(EPS)

S1 Table Hazard ratio estimates for multivariable models. All HR estimates from multivariable Cox proportional hazards models on time to post-biopsy BCR.
(DOCX)

S2 Table Hazard ratios in univariable models. All HR estimates from univariable Cox proportional hazards models on time to post-prostatectomy BCR. These results are essentially the same as for BCR calculated from time of biopsy (see Table 2).
(DOCX)

S3 Table Hazard ratios for multivariable models. HR estimates from multivariable Cox proportional hazards models involving the GPS result on time to post-prostatectomy BCR (n = 120 for models 1, 4; n = 119 for models 2,3,5,6). These results are essentially the same as for BCR calculated from time of biopsy (see Table 2).
(DOCX)
