# Supplementary material for: The 17-gene Genomic Prostate Score assay as a predictor of biochemical recurrence in men with intermediate and high-risk prostate cancer
Source: PLoS One. 2022 Sep 1;17(9):e0273782. doi: 10.1371/journal.pone.0273782 (PMC9436076; doi:10.1371/journal.pone.0273782)
Supplement: S1 Table — All HR estimates from multivariable Cox proportional hazards models on time to post-biopsy BCR. (DOCX) [file pone.0273782.s005.docx]

**S1 Table.** Multivariable Cox proportional hazards models on time to post-biopsy BCR in patients with unfavorable intermediate or higher risk prostate cancer (n = 120).

| **Model** | | **Variable** | **Events / N** | **HR** | **95% CI** | **p-value** |
| --- | --- | --- | --- | --- | --- | --- |
| 1 | | GPS result per 20-unit increase | 35 / 120 | 2.14 | 1.31 to 3.46 | 0.003 |
|  | | NCCN risk group |  |  |  | 0.021 |
|  | | Unfavorable Intermediate | 22 / 88 | 1.00 (ref) |  |  |
|  | | High | 9 / 27 | 1.92 | 0.83 to 4.09 |  |
|  | | Very High | 4 / 5 | 5.48 | 1.51 to 15.62 |  |
| 2 | | GPS result per 20-unit increase | 35 / 119 | 2.28 | 1.35 to 3.82 | 0.002 |
|  | | Diagnostic PSA (ng/mL) per one SD increase | 35 / 119 | 1.57 | 1.21 to 1.99 | 0.002 |
|  | | Biopsy Grade Group |  |  |  | 0.099 |
|  | | 2 | 9 / 51 | 1.00 (ref) |  |  |
|  | | 3 | 16 / 45 | 2.25 | 0.98 to 5.56 |  |
|  | | 4 | 10 / 23 | 2.39 | 0.89 to 6.53 |  |
| 3 | | GPS result per 20-unit increase | 35 / 119 | 2.15 | 1.29 to 3.57 | 0.004 |
|  | | PSA density (ng/mL^2^) per one SD increase | 35 / 119 | 1.55 | 1.21 to 1.91 | 0.001 |
|  | | Biopsy Grade Group |  |  |  | 0.065 |
|  | | 2 | 9 / 51 | 1.00 (ref) |  |  |
|  | | 3 | 16 / 45 | 2.07 | 0.91 to 5.13 |  |
|  | | 4 | 10 / 23 | 2.92 | 1.13 to 7.78 |  |
| 4 | | GPS result (dichotomous) |  |  |  | 0.003 |
|  | | 0-40 | 10 / 64 | 1.00 (ref) |  |  |
|  | | 41-100 | 25 / 56 | 3.00 | 1.43 to 6.72 |  |
|  | | NCCN risk group |  |  |  | 0.020 |
|  | | Unfavorable Intermediate | 22 / 88 | 1.00 (ref) |  |  |
|  | | High | 9 / 27 | 1.97 | 0.85 to 4.20 |  |
|  | | Very High | 4 / 5 | 5.31 | 1.50 to 14.84 |  |
| 5 | | GPS result (dichotomous) | 35 / 119 |  |  | 0.005 |
|  | | 0-40 | 10 / 63 | 1.00 (ref) |  |  |
|  | | 41-100 | 25 / 56 | 2.84 | 1.36 to 6.35 |  |
|  | | Diagnostic PSA (ng/mL) per one SD increase | 35 / 119 | 1.55 | 1.18 to 1.98 | 0.003 |
|  | | Biopsy Grade Group |  |  |  | 0.174 |
|  | | 2 | 9 / 51 | 1.00 (ref) |  |  |
|  | | 3 | 16 / 45 | 2.07 | 0.90 to 5.13 |  |
|  | | 4 | 10 / 23 | 2.15 | 0.77 to 6.04 |  |
| 6 | | GPS result (dichotomous) |  |  |  | 0.003 |
|  | | 0-40 | 10 / 63 | 1.00 (ref) |  |  |
|  | | 41-100 | 25 / 56 | 3.00 | 1.44 to 6.70 |  |
|  | | PSA density (ng/mL^2^) per one SD increase |  | 1.61 | 1.25 to 1.98 | <.001 |
|  | | Biopsy Grade Group |  |  |  | 0.121 |
|  | | 2 | 9 / 51 | 1.00 (ref) |  |  |
|  | | 3 | 16 / 45 | 1.91 | 0.83 to 4.77 |  |
|  | | 4 | 10 / 23 | 2.61 | 1.00 to 7.00 |  |
|  | BCR = biochemical recurrence; GPS = Genomic Prostate Score; NCCN = National Comprehensive Cancer Network; PSA = prostate-specific antigen; ref = reference group; RP = radical prostatectomy. | | | | | |
