# Supplementary material for: The 17-gene Genomic Prostate Score assay as a predictor of biochemical recurrence in men with intermediate and high-risk prostate cancer
Source: PLoS One. 2022 Sep 1;17(9):e0273782. doi: 10.1371/journal.pone.0273782 (PMC9436076; doi:10.1371/journal.pone.0273782)
Supplement: S2 Table — All HR estimates from univariable Cox proportional hazards models on time to post-prostatectomy BCR. These results are essentially the same as for BCR calculated from time of biopsy (see Table 2). (DOCX) [file pone.0273782.s006.docx]

**S2 Table.** Univariable Cox proportional hazards models results on time from radical prostatectomy to BCR (n = 120). These results are essentially the same as models on time from biopsy to BCR (see Table 2).

| **Variable** | **Events / N** | **HR** | **95% CI** | **p-value** |
| --- | --- | --- | --- | --- |
| GPS result |  |  |  |  |
| GPS result per 20-unit increase | 35 / 120 | 2.33 | 1.43 to 3.76 | <.001 |
| GPS result |  |  |  | <.001 |
| 0-40 | 10 / 64 | 1.00 (ref) |  |  |
| 41-100 | 25 / 56 | 3.28 | 1.62 to 7.18 |  |
| Clinical/demographic/pathologic: continuous^a^ |  |  |  |  |
| Age (yrs) at diagnosis | 35 / 120 | 1.19 | 0.83 to 1.76 | 0.356 |
| BMI (kg/m^2^) | 35 / 120 | 0.80 | 0.55 to 1.13 | 0.213 |
| Diagnostic PSA (ng/mL) | 35 / 120 | 1.55 | 1.20 to 1.91 | 0.002 |
| % positive cores | 35 / 120 | 1.16 | 0.82 to 1.65 | 0.406 |
| PSA density (ng/mL^2^) | 35 / 120 | 1.58 | 1.23 to 1.94 | <.001 |
|  |  |  |  |  |
| Clinical/demographic/pathologic: categorical |  |  |  |  |
| Age (yrs) at diagnosis |  |  |  | 0.177 |
| < 65 | 15 / 64 | 1.00 (ref) |  |  |
| ≥ 65 | 20 / 56 | 1.58 | 0.81 to 3.15 |  |
| BMI (kg/m^2^) |  |  |  | 0.680 |
| < 25 | 6 / 16 | 1.00 (ref) |  |  |
| 25 - < 30 | 7 / 29 | 0.60 | 0.20 to 1.91 |  |
| ≥ 30 | 22 / 75 | 0.74 | 0.32 to 2.02 |  |
| Biopsy grade group^b^ |  |  |  | 0.021 |
| 2 | 9 / 51 | 1.00 (ref) |  |  |
| 3 | 16 / 45 | 2.21 | 0.97 to 5.44 |  |
| 4 | 10 / 23 | 3.56 | 1.40 to 9.33 |  |
| Clinical stage |  |  |  | 0.735 |
| T1c | 26 / 91 | 1.00 (ref) |  |  |
| T2a | 3 / 14 | 0.73 | 0.17 to 2.09 |  |
| T2b/c | 6 / 15 | 1.26 | 0.47 to 2.88 |  |
| NCCN risk group |  |  |  | 0.005 |
| Unfavorable Intermediate | 22 / 88 | 1.00 (ref) |  |  |
| High | 9 / 27 | 1.79 | 0.78 to 3.79 |  |
| Very High | 4 / 5 | 8.62 | 2.46 to 23.48 |  |
| PSA density (ng/mL^2^) |  |  |  | 0.371 |
| ≥ 0.15 | 5 / 22 | 1.00 (ref) |  |  |
| < 0.15 | 30 / 98 | 1.51 | 0.64 to 4.44 |  |
|  |  |  |  |  |
| ^a^ HR is for a one standard deviation (SD) increase. See Table 1 for SD values.  ^b^ n=119, excluding one patient with biopsy Grade Group 1.    BMI = body mass index, GPS = Genomic Prostate Score, HR = hazard ratio, PSA = prostate-specific antigen. | | | | |
