# Supplementary material for: The 17-gene Genomic Prostate Score assay as a predictor of biochemical recurrence in men with intermediate and high-risk prostate cancer
Source: PLoS One. 2022 Sep 1;17(9):e0273782. doi: 10.1371/journal.pone.0273782 (PMC9436076; doi:10.1371/journal.pone.0273782)
Supplement: S3 Table — HR estimates from multivariable Cox proportional hazards models involving the GPS result on time to post-prostatectomy BCR (n = 120 for models 1, 4; n = 119 for models 2,3,5,6). These results are essentially the same as for BCR calculated from time of biopsy (see Table 2). (DOCX) [file pone.0273782.s007.docx]

**S3 Table.** Multivariable Cox proportional hazards models results on time from radical prostatectomy to BCR (n = 120 for models 1, 4; n=119 for models 2,3,5,6). These results are essentially the same as models on time from biopsy to BCR (see Table 3).

| **Model** | **HR for GPS** | **95% CI** | **p-value** |
| --- | --- | --- | --- |
| **Continuous GPS result** |  |  |  |
| 1: GPS result per 20-unit increase, NCCN risk group | 2.11 | 1.29 to 3.42 | 0.003 |
| 2: GPS result per 20-unit increase, diagnostic PSA, biopsy GS | 2.22 | 1.32 to 3.72 | 0.003 |
| 3: GPS result per 20-unit increase, PSA density, biopsy GS | 2.14 | 1.28 to 3.55 | 0.004 |
| **Dichotomous GPS result** |  |  |  |
| 4: GPS result 41-100 vs 0-40), NCCN risk group | 3.00 | 1.44 to 6.68 | 0.003 |
| 5: GPS result (41-100 vs 0-40), diagnostic PSA, biopsy GS | 2.86 | 1.38 to 6.34 | 0.004 |
| 6: GPS result (41-100 vs 0-40), PSA density, biopsy GS | 3.01 | 1.46 to 6.68 | 0.003 |

| BCR = biochemical recurrence; GPS=Genomic Prostate Score; GS = Gleason Score; NCCN = National Comprehensive Cancer Network; PSA = prostate-specific antigen; RP = radical prostatectomy. |
| --- |
